# Impact of Attention-Deficit/Hyperactivity Disorder (ADHD) on prescription dug spending for children and adolescents: increasing relevance of health economic evidence

**DOI:** 10.1186/1753-2000-1-13

**Published:** 2007-11-15

**Authors:** Michael Schlander

**Affiliations:** 1 Institute for Innovation & Valuation in Health Care (INNOVAL^HC^), Eschborn, Germany; 2 University of Applied Economic Sciences Ludwigshafen, Germany; 3 Mannheim Medical Faculty, University of Heidelberg, Germany

## Abstract

**Background:**

During the last decade, pharmaceutical spending for patients with attention-deficit-hyperactivity disorder (ADHD) has been escalating internationally.

**Objectives:**

First, to estimate future trends of ADHD-related drug expenditures from the perspectives of the statutory health insurance (SHI; Gesetzliche Krankenversicherung, GKV) in Germany and the National Health Service (NHS) in England, respectively, for children and adolescents age 6 to 18 years. Second, to evaluate the budgetary impact on individual prescribers (child and adolescent psychiatrists and pediatricians treating patients with ADHD) in Germany.

**Methods:**

A model was developed to predict plausible scenarios of future pharmaceutical expenditures for treatment of ADHD. Model inputs were derived from demographic and epidemiological data, a literature review of past spending trends, and an analysis of new pharmaceutical products in development for ADHD. Only products in clinical development phase III or later were considered. Uncertainty was addressed by way of scenario analysis. For each jurisdiction, five scenarios used different assumptions of future diagnosis prevalence, treatment prevalence, rates of adoption and unit costs of novel drugs, and treatment intensity.

**Results:**

Annual ADHD pharmacotherapy expenditures for children and adolescents will further increase and may exceed €310 m (D; E: ₤78 m) in 2012 (2002: ~€21.8 m; ~₤7.0 m). During this period, overall drug spending by individual physicians may increase 2.3- to 9.5-fold, resulting from the multiplicative effects of four variables: increased number of diagnosed cases, growing acceptance and intensity of pharmacotherapy, and higher unit costs of novel medications.

**Discussion:**

Even for an extreme low case scenario, a more than six-fold increase of pharmaceutical spending for children and adolescents is predicted over the decade from 2002 to 2012, from the perspectives of both the NHS in England and the GKV in Germany. This budgetary impact projection represents a partial analysis only because other expenditures are likely to rise as well, for instance those associated with physician services, including diagnosis and psychosocial treatment. Further to this, by definition budgetary impact analyses have little to nothing to say about clinical appropriateness and about value of money.

**Conclusion:**

Providers of care for children and adolescents with ADHD should anticipate serious challenges related to the cost-effectiveness of interventions.

## Background

In the United States, identification of attention-deficit/hyperactivity disorder (ADHD) among children and adolescents showed the greatest increase of all categories of psychosocial problems [1], and the percentage of children with a diagnosis of ADHD receiving medications increased from 32% in 1979 to 78% in 1996 [1-3]. For the mid-1990s, overall psychostimulant treatment prevalence rates in children and adolescents were found at about 2.5% [4], ranging from 1.9% in a California health plan [5] to 3.0% in national database analyses [6,7]. In the late 1990s, growth rates of stimulant prescriptions accelerated in the United States [8,9]. For 1997, treatment prevalence was reported at 4.1% (or 78% of children with ADHD) [10]. In a survey of school nurses conducted in Baltimore, Maryland, in 1998, 2.92% of all public school students (N = 816,465) were administered a medication for ADHD in school; 84% of those (2.46%) received methylphenidate [4]. In 1999, in a nationally representative, commercially insured sample population 5 to 14 years old, the one-year prevalence of stimulant treatment was 4.2 percent in the United States [11]. According to another survey conducted between 1997 and 1999 among parents of elementary school children in public elementary schools in North Carolina, medication prevalence was even 7% in the population studied, with stimulants accounting for 93% of the prescriptions [12]. More recent data have been somewhat contradictory, with one analysis finding a treatment prevalence of 4.8% in children (age 6–12 years) and 3.2% in adolescents (age 13–19 years), suggesting that the steep increase of the late 1990s may have attenuated in these age groups [13], whereas another study published by Medco Health Solutions [14], a large U.S. pharmacy benefit management organization, still found the number of individuals age 19 years or younger using ADHD medications increasing by 49% (males) and 82% (females) between 2000 and 2004.

**Figure 1:**
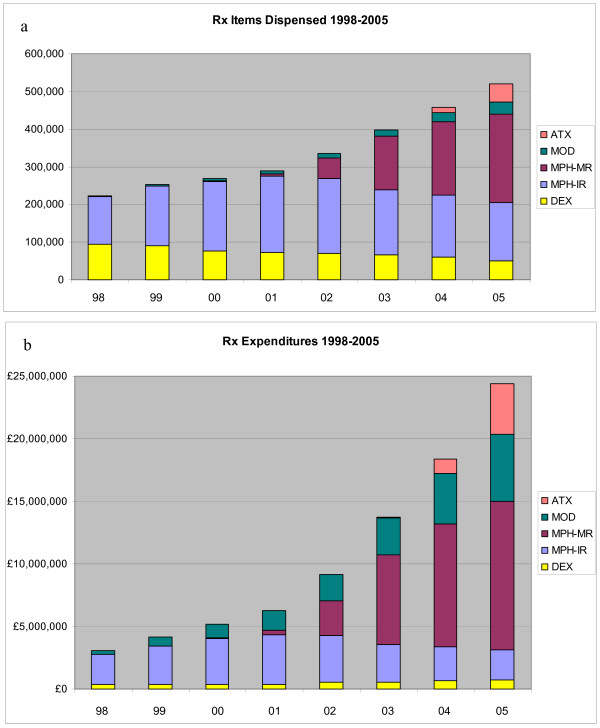
**ADHD-related prescriptions and expenditures in England, 1998 – 2005**. a: Prescription items dispensed in the community; b: Expenditures by category p.a.; DEX: dexamphetamine (Dexedrine^R ^and others); MPH: methylphenidate; IR: immediate-release formulations (Ritalin^R ^and generics); MR: modified-release formulations (Concerta^R ^XL, Equasym^R ^XL; Ritalin^R ^SR imports); MOD: modafinil (Provigil^R^, licensed for daytime sleepiness); ATX: atomoxetine (Strattera^R^); PEM: pemoline (Volital^R^, before 2002 only, not shown due to small volume); data source: NHS Prescription Cost Analysis 1999–2006 [19]. Note that these data include prescriptions for adults with ADHD and also for other indications (narcolepsy).

**Figure 2:**
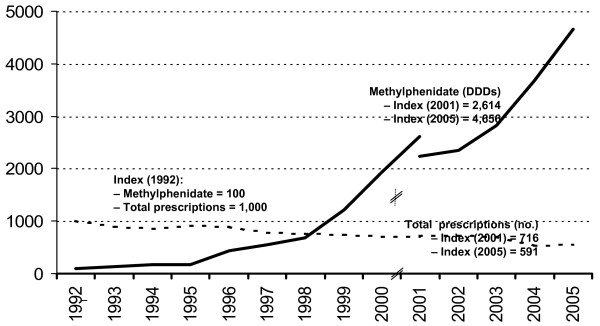
**Methylphenidate prescriptions trend in Germany, 1992 – 2005**. Methylphenidate prescriptions grew 47-fold from 1992 to 2005. During the same period, total prescriptions in Germany declined by 41 percent. Data source: Wissenschaftliches Institut der AOK, Schwabe and Paffrath, 1993 – 2006 [20]; note change of database for year 2001/2002. All data refer to prescriptions reimbursed by statutory health insurance (SHI, "GKV", covering approximately 90 percent of German population); excluding parallel imports. Note that these data include prescriptions for adults with ADHD and also for other indications (narcolepsy).

Meanwhile, the growth of pediatric psychotropic prescriptions has become an international phenomenon [15]. For stimulant treatment, similar trends as in the US – albeit at lower absolute levels – have been observed in Canada [16] and Europe. For instance, in Spain the consumption of methylphenidate increased by 8% annually from 1992 to 2001 [17]. A study in six general practices in the Netherlands showed a threefold rise of methylphenidate users between 1998 and 2003 [18]. In England, the number of methylphenidate prescriptions dispensed in the community increased from 126,000 in 1998 to 389,200 in 2005 (or +207%; cf. Figure 1a), compared to a total growth of prescriptions of +40% during the same period [19]. In Germany, the number of defined daily doses (DDDs) of methylphenidate prescribed for outpatients insured by statutory sick funds ("Gesetzliche Krankenversicherung", GKV, covering approximately 90% of the German population) grew 47-fold between 1992 and 2005 [20], while total prescriptions written in Germany decreased by 41% in the same period (cf. Figure 2).

Much of the debate triggered by this trend has focused on medical aspects, such as concerns about potential overuse of psychostimulants [21,22] and adverse treatment effects [23]. It has, however, been argued that underuse may be more of a problem, at least in Europe [24], and low rates of recognition of ADHD or hyperkinetic disorder in the United Kingdom were associated with insensitive diagnostic assessments [25]. Recent German methylphenidate prescription analyses based on a regional sample of 11,235 children and adolescents with a diagnosis of ADHD in Germany (year 2003) did not reveal overuse [26]. Even for the United States, no evidence was found to substantiate claims of abuse and inappropriate use of methylphenidate [27], although there have been occasional reports on diversion to peers of stimulant medications, especially short-acting formulations [28,29].

Beyond clinical implications, in an era of limited available resources, the economic dimension associated with increased health care utilization can no longer be ignored. In economic terms, the opportunity cost of medical interventions will be approximated by their budgetary impact – in particular, if a payers' perspective is adopted. Indeed, budgetary impact analyses are requested by a growing number of health care policy makers as an input into the decision-making process about health care resource allocation [30]. In practice, results of such analyses are frequently interpreted with a notion of "affordability" in mind. Unfortunately budget impact may exceed the pure effects of increased treatment rates if, for instance, rates of diagnosis (administrative prevalence), unit costs (for instance owing to introduction of novel, usually more expensive treatment options, which may be used in addition to or substituting cheaper alternatives), or treatment intensity (in terms of dose or duration) change simultaneously. No doubt, all these factors come into play in ADHD among children and adolescents. More than this, they may interact with each other, reinforcing their combined effects: for instance, availability of more expensive novel medications will result in enhanced communication and promotional efforts by their manufacturers, which in turn will – in addition to their direct influence on physician treatment choices (i.e., increase market share) – influence awareness and identification of the disorder (i.e., increase market size and aggregate number of prescriptions [25,31,32]). Furthermore, recent results of long-term clinical trials such as the landmark NIMH Multimodal Treatment Study (MTA) have demonstrated the effectiveness, in particular in terms of ADHD core symptom relief, of an intense medication management strategy [33-38]), thus contributing to the increasing acceptance and intensity of pharmacotherapy.

With the introduction of new products commanding higher units costs (cf. Table 1), growth of pharmaceutical spending has outpaced the rising number of prescriptions. In the United States, prescription drug expenditures for ADHD in children increased by 183% between 2000 and 2003. By 2003, spending on behavioral medications to treat children had overtaken both the antibiotic and asthma segments, which are traditionally high-use categories in pediatric medicine [39]. According to IMS data, total U.S. sales of ADHD drugs reportedly rose from US-$759 million in 2000 to US-$3.1 billion in 2004 [40]. Analysts from Merrill Lynch expect the U.S. ADHD market (including adult patients) to exceed US-$4 billion by 2010 on the back of *new products *(cf. below, Table 2[41]).

**Table 1:** Treatment options for ADHD in children and adolescents in Germany (D) and the United Kingdom (UK): product availability and acquisition cost

**Active compound**	**Formulation**	**Abbreviation**	**Trade name**	**Manufacturer**	**Authorization^1^**	**Cost per treatment day^2^**		
					UK/D	UK	D	Assumption
Dexamphetamine sulphate	Tablets (5 mg)	DEX	Dexedrine^R^	UCB (Celltech)	≤ 2000	£ 0.43	n.a.	20 mg/d
Methylphenidate hydrochloride	Immediate-release tablets (10 mg)	MPH-IR-o	Ritalin^R^	UK: Cephalon; D: Novartis	≤ 2000	£ 0.56	€1.58	30 mg/d (DDD), divided in three daily doses
Methylphenidate hydrochloride	Immediate-release tablets (5, 10, 20 mg)	MPH-IR-b	Branded Generics: Equasym^R^Medikinet^R^	UCB (previously Celltech); Medice (D only)	≤ 2000	£ 0.52	€1.41	30 mg/d (DDD), divided in three daily doses
Methylphenidate hydrochloride	Immediate-release tablets (5, 10, 20 mg)	MPH-IR-g	Generics (misc. non-proprietary)	1A, TAD, et al.		£ 0.38	€1.13	30 mg/d (DDD), divided in three daily doses
Methylphenidate hydrochloride	Modified-release tablets (18, 36, 54 mg)	MPH-MR12	Concerta^R ^XL (OROS delivery system)	Janssen-Cilag	UK: 2002 (Feb., 19)	£ 1.23	€2.94	36 mg/d (administered once daily)
Methylphenidate hydrochloride	Modified-release capsules (10, 20, 30, 40 mg)	MPH-MR08 (note different formulations)	Equasym^R ^XL (biphasic Diffucaps delivery system);	UCB (previously Celltech);	UK: 2005 (Feb., 11); D: mutual recognition May 2006	£ 1.17		30 mg/d (administered once daily)
			Medikinet^R ^retard	Medice (D only)	D: January 2005		€2.46	
Atomoxetine hydrochloride	Hard capsules (10, 18, 25, 40, 60 mg)	ATX	Strattera^R^	Eli Lilly	UK: 2004 (May, 27); D: May 2005	£ 1.95 £ 3.80	€3.88 €7.76	(once daily. administration) (administration divided in two daily doses)

^1^First authorization in UK, from electronic Medicines Compendium, available online at , accessed August 12, 2005.

^2^NHS acquisition costs (not accounting for negotiated procurement discounts in some settings), taken from British National Formulary 51, March 2006 [75]; note that individual doses and thus costs may vary. German data retrieved from "Gelbe Liste", July 2006 [76]. Note that, at the time of printing this paper, in Germany reference prices have been proposed for products containing methylphenidate as the active ingredient.

**Table 2:** Data sources and assumptions

**(1) Demographic data**
*Basic demographic data *came from national bureaus [77,78]. As a model input, rounded figures of 9.72 million (for Germany, 90% of 10.8 million, reflecting population coverage by the GKV) and 8.1 million (England; with the NHS covering the total population) were used for the number of persons age 6–18 years. As an estimate of the true prevalence of ADHD, 6% was used for base case analysis (cf. below, scenarios) assuming that DSM-IV criteria [79] will be used in clinical practice. A sample of pediatricians surveyed in the German region of Nordbaden [74] had suggested that ADHD was diagnosed according these criteria, instead of ICD-10 [80] criteria often referred to in the European literature [81]. The figure of 6% came from reviews of epidemiological studies [82,83] that coincided with findings from a German mail survey of 165 parents of children age 6 to 10 years using a parent rating scale for ADHD [84]. The British Child and Adolescent Mental Health Survey described a prevalence of 2.23% (1999); its authors noted that diagnoses might be missed if information is not sought from teachers about children's functioning in school [85].
**(2) ADHD diagnosis rates**
Information on *ADHD recognition rates *could be obtained from two regional studies: A claims data analysis using a sample of 35,000 children age 3 to 15 years covered by a regional sick fund (AOK) in Hessen, Germany, indicated that the administrative prevalence of ADHD increased from 1.24% in 1998 to 2.43% in 2001 [86]. In 2003, the administrative prevalence of ADHD among children and adolescents age 7 to 19 years in the German region of Nordbaden (N = 317,520) was 2.95% [74]. In line with historic trends up to 2005, it was assumed that awareness of ADHD would continue to increase and result in recognition rates going up from approximately 50% (or 3% of the total age group) in 2003 to plateau at 70–80% or 4.5% of the population aged 6–18 years from 2010 onwards (for England 65(-70)%, in line with current trends).
**(3) ADHD treatment rates**
Data on the *rate of patients receiving drug therapy *(i.e., treatment prevalence) were derived from the regional analyses and tested for consistency with top-down estimates based on the number of prescriptions dispensed, revenues booked, and assumed treatment intensity – cf. (6), below. These top-down calculations included adjustment for an assumed 5% share of psychostimulant prescriptions for narcolepsy, gradually declining as ADHD prescriptions rise. They were also adjusted for an estimated 10% off-label prescriptions for adult patients with ADHD in 2003, as indicated by new data from the Nordbaden project [75]. – Analyses of the AOK Hessen sample (for year 2000) revealed a methylphenidate treatment prevalence of 0.52% among children and adolescents below age 20 [87]. Of children age 6 to 15 years with a diagnosis of ADHD in the AOK Hessen sample, 17% were prescribed methylphenidate in 1998; and this rate increased to 29% in 2000 ([88], p. 32). More recent data from Nordbaden indicate that in 2003, 40% (1,161 out of 2,939) of children and adolescents (age group 7–19) with a diagnosis of ADHD were treated with stimulant medication [74,89]. For modeling input, these treatment rates were adjusted for regional variation: In 2001, methylphenidate prescriptions (defined daily doses, DDDs, per population below age 20) were 17% below the national average in Hessen, while they were 23% above the average in Nordbaden [90]. Further it was assumed that the rate of ADHD patients receiving pharmacotherapy would continue to increase through 2010 (and remain stable thereafter), reflecting mounting evidence of long-term treatment effectiveness (cf. earlier, *Introduction *[33-37]), a growing number of alternative treatment options (cf. below), and the communication efforts by manufacturers competing for market share. Model inputs for England were derived from top-down calculations, corrected for prescriptions for adult ADHD patients; regarding narcolepsy, it was assumed that this indication would be covered by modafinil (trade name Provigil^R^), which has been licensed for the treatment of daytime sleepiness associated with narcolepsy or obstructive sleep apnoea [75]. – Note that due to these adjustments model estimates for years 2001 to 2005 deviate from *total *market data delineated in the *Introduction*.
**(4) New product profiles and availability**
An extensive literature and database search was conducted to obtain information on the *expected availability of new products and their likely therapeutic profiles*. In addition to standard Medline searches, presentations at recent psychiatry and child and adolescent psychiatry congresses in the United States and Europe were screened for reports on ADHD treatment. To identify new products on the horizon, further research on drug development programs in the field was conducted using the pharmaceutical databases of Scrip World Pharmaceutical News and Therapeutics-Daily [49,61]. For key findings, see *Results*, below, and Table 3. As a rule, it was assumed that new products would become available in Europe (with no difference between Germany and England) 18–24 months later than in the United States, reflecting the predominant strategy of pharmaceutical companies to choose the United States (currently accounting for more than 90% of global ADHD drug sales) as lead market [49].
**(5) Market diffusion rates**
*Diffusion rates and market shares *were modeled separately for each alternative preparation, both by category and by product. Each of the quantitative model inputs was informed by findings of the literature and database review (cf. above, 4, and *Results*, below), and supported by expert consensus derived from semi-structured interviews with experienced pharmaceutical market specialists. For immediate-release preparations of methylphenidate (MPH-IR), generic market penetration was assumed to reach 70–75% towards the end of the projection period (branded MPH-IR, Ritalin^R^, 10%, and dexmethylphenidate, Focalin^R^, 20% – cf. also *Results*); this applied similarly to the projections for England and Germany.
**(6) Treatment intensity**
*Treatment intensity *was expressed by a single index representing the average number of days on treatment with one defined daily dose (DDD) per day. (For atomoxetine, in a certain number of patients twice daily dosing may be required [91,92]; this was assumed to be the case in 20% of patients on this drug.) On the basis of a methylphenidate (MPH-IR) prescribing analysis in the German region of Hessen, it was estimated that the median duration of treatment had been about 120 days in 2000 [87]. This figure may underestimate the actual duration of treatment since no adjustment for data edge effects was made in this study. Treatment persistence with modified-release products should be higher owing to improved treatment compliance. This expectation has received support from two independent Medicaid claims data studies from California and Texas that found a 37% increase in uninterrupted duration of initial MPH-MR treatment compared to MPH-IR [93,94]. Between 2000 and 2003, mean duration of ADHD treatment was 158 days with MPH-MR and 128 days with MPH-IR [93]. It was further assumed for some scenarios (cf. below, Table 3) that average treatment intensity would tend to increase reflecting findings of the NIMH MTA study [35-37].
**(7) Product acquisition costs**
*Acquisition costs *per defined daily dose were calculated for each product from the perspectives of the NHS (England) or the GKV (Germany), respectively. For marketed products, data for large pack sizes (Germany: N2, typically containing 28 to 30 single doses) were retrieved for March 2006 (England) or July 2006 (Germany) from standard sources [75,76]. Ex-pharmacy prices were not corrected for co-payments since the vast majority of patients age 18 years or younger have been exempt from cost-sharing in both England and Germany. It was generally assumed that there would be no price increases during the projection period. Pricing assumptions for new products are described below (cf. also Tables 1 and 3).
**(8) Prescribing patterns**
Data indicating *physician-specific prescription patterns *were available for Germany only. At the same time, such data may be most relevant to German physicians, since they are subject to individual drug budget regulation, which differs from the system of Primary Care Groups in England [95]. Pediatricians treated most (5,605 of 11,245 below age 20 years, or 50%) of these patients in Nordbaden (2003), followed by child# and adolescent psychiatrists (3,369 or 30%) [74]. For comparison, in the smaller Hessen sample from 1998–2000, pediatricians accounted for 44% of methylphenidate prescriptions in 2000 [90]. Within physician groups, care for patients with ADHD was highly concentrated in Nordbaden: the top-50% of child and adolescent psychiatrists accounted for 92.1% of ADHD patients treated by their group, and the top-20% of pediatricians accounted for a share of 66.2%. Since budgetary impact of ADHD medications will be of relevance only to those physicians involved in care for these patients, the average impact was determined for the top-50% of child and adolescent psychiatrists and the top-20% of pediatricians, respectively. Total prescription drug spending caused by pediatricians (including drugs for ADHD) was €92,000 per physician in 2004 (2002: €83,000) [20]. Total drug spending of each of the top-50% child and adolescent psychiatrists was estimated at €51,000 in 2004, assuming that 76% of their psychotropic drug expenditures in this year were due to ADHD treatment, derived from data on psychotropic prescriptions for children age 15 or younger [20]. This estimate was consistent with prescribing data obtained from Nordbaden [96], when allowing for regional variance described above. Further assuming non-ADHD expenditures to grow at an annual rate of 5%, the impact of projected future ADHD spending on individual physicians could be estimated.

In Germany, total GKV outpatient spending for psychostimulants rose from €1.25 m in 1995 to €23.7 m in 2002 (before the first modified-release product had been launched) and €51.6 million in 2004 [20], 56% of which were accounted for by MPH-MR12. Spending for ADHD medications reached €82 million in 2005, exceeding our earlier forecasts, which had predicted annual expenditures of approximately €108 million (range from €62 to €155 million) by 2009 [42,43]. Apart from the launch of atomoxetine in March 2005, this substantial increase was primarily driven by spending for two modified-release preparations of methylphenidate (cf. Table 1), both of which ranked among the top-100 products in terms of sales [44].

In England spending for ADHD-related pharmacotherapy, including modafinil and atomoxetine, increased from 1998 (₤3.1 million) to 2005 (₤24.4 million) by +695% (Figure 1b), again exceeding the growth of prescriptions (for ADHD treatments between 1998 and 2005, +132%)[19]. One new product (MPH-MR12, cf. Table 1) alone accounted for 42% of prescriptions, and 45% of pharmaceutical expenditures, for ADHD in 2005 [19]. Average costs per prescription rose steadily from ₤13.68 in 1998 to ₤46.94 in 2005, driven by price increases for dexamphetamine and a shift to more expensive new products: Combined, all new products with once-daily administration (in principle, MPH-MR08, MPH-MR12, Ritalin SR, and ATX) accounted for 54% of total ADHD-related prescriptions and 65% of sales in 2005 [19]. These Prescription Cost Analysis data for England do not include items dispensed in hospitals.

The high rate of adoption of new products with once-a-day administration schedules reflects the well-known problems associated with administration of a mid-day dose (during school) in children and adolescents with ADHD [45,46]. Obviously, here a disorder-specific clinical need contributes to the fact that current trends of ADHD prescribing behavior mirror a more general pattern encountered in European pharmaceutical markets, namely, that the diffusion of new products consistently represents the single most important growth driver [47]. These observations raise the urgent question whether, and to what extent, pharmaceutical spending for children and adolescents with ADHD may continue to escalate in the foreseeable future. It is the purpose of this paper to shed some light on this issue, using the examples of England and Germany.

## Methods

A forecasting model was developed to project the likely pharmaceutical expenditures for children and adolescents (age 6–18 years) with a diagnosis of ADHD in England and Germany through 2012, specifying assumptions and assumed relationships between variables in a transparent manner. Model outcomes were used to estimate the impact on drug spending by individual German physicians involved in care for ADHD patients, after validation of the model by replicating historic data (from 1998 to 2005). Key uncertainties were addressed using scenario analysis, combining objective information with specific assumptions about future events [48].

The model was restricted to pharmaceutical spending. It combined, in a hierarchical structure, (1) epidemiological information (demographic and prevalence data) with assumptions on (2) recognition rates (diagnosis prevalence), (3) rate of patients receiving drug therapy (treatment prevalence), (4) availability and adoption of new products, incorporating information on therapeutic profiles, (5) diffusion and market shares for alternative preparations, by category and by product, including generic substitution, (6) treatment intensity (expressed as average number of days times defined daily doses), (7) acquisition cost per defined daily dose for each product, from the perspectives of the NHS (England) or the GKV (Germany), respectively. For validation of the model, available data on the variables above were used to compare model outcomes with the historic evolution of drug spending in England and Germany. Extensions of the model were used to estimate the impact of the projections on drug budgets of individual pediatricians and specialists in child and adolescent psychiatry participating in care for patients with ADHD in Germany. Details on data sources and assumptions are provided in Table 2.

## Results

### 1. New products in development (and further assumptions)

The market for ADHD treatment has attracted pharmaceutical companies to invest heavily in new product development. Results of literature searches and the database analysis [49] are summarized in Table 3.

**Table 3:** New products in development for treatment of ADHD in children and adolescents: overview of compounds not yet available in England and Germany

**Active ingredient**	**Abbreviation/Pharmaceutical preparation**	**Trade name (US)**	**Manufacturer**	**Approval status (US)**	**Notes**
Methylphenidate hydrochloride	MPH-MR08 (modified-release preparation)	Ritalin^R ^LA (using SODAS delivery system developed by Elan)	Novartis	available in US and Switzerland	
Methylphenidate hydrochloride	MPH-TDS Patch for transdermal drug delivery (o.a.d); 12 h duration of action	Daytrana^R ^DOT matrix transdermal technology	Shire (in license from Noven)	US approval (children age 6–12 years) April 2006; 2nd line to oral drugs	Skin sensitization reported in 13–22% of subjects wearing the patch; product had been deemed non-approvable by FDA before (April 2003)
Dexmethylphenidate, a non-racemic form of methylphenidate:	d-MPH (the active isomer of methylphenidate)	Focalin^R^	Novartis (in license from Celgene)	Approved in US	
Dexmethylphenidate, a non-racemic form of methylphenidate:	d-MPH-ER (extended release formulation)	Focalin^R ^XR	Novartis (in license from Celgene)	US approval (for "children, adolescents, and adults") May 2005	
Lisdexamphetamine dimesylate	LisDEX; (pharmaceutical preparation with a o.a.d. dosing schedule)	NRP104	Shire (in license from New River Pharmaceuticals)	US approval (for children age 6–12 years) granted in 2007	Reduced abuse potential expected because amphetamine is linked to L-lysine and does no become active until metabolized in the gastrointestinal tract
Mixed amphetamine salts	MAS (immediate and extended release formulations	Adderall^R^, Adderall^R ^XR	Shire	Available in US	Unlikely to be approved in Europe
Modafinil Successor compound: Armodafinil	MOD; dopamine reuptake inhibitor; effects on neuropetides possible ARM	Sparlon^R ^(licensed in US and UK as Provigil^R ^for narcolepsy) Nuvigil^R^	Cephalon (Sparlon^R ^was planned to be co-promoted in the US by McNeil, a sister company of Janssen-Cilag)	After receiving a non-approvable letter for modafinil in ADHD from the FDA in August 2006, Cephalon refocused its R&D on armodafinil [61]	Suspected serious adverse events (skin rash/Stevens-Johnson syndrome) in association with modafinil

Only projects in phase III of clinical development or products already marketed in the United States. Daytrana^R ^was formerly known as "Methypatch^R^", Sparlon^R ^as "Attenace^R^". Data source: InnoVal-HC, 2006 [49].

A number of further pharmaceutical preparations and variants of methylphenidate are in phase III of clinical development in Europe, some of which have already received marketing authorization in the United States. These include a transdermal system (TDS, approved as Daytrana^R ^in the United States, April 2006) of methylphenidate with duration of action of 12 hours (wear time, 9 hours). At present, in the absence of head-to-head trials against established oral formulations, the main advantage of this product seems to be convenience-related [49-51]. For modeling, it has therefore been assumed that it would capture no more than 10% of methylphenidate prescriptions. This implies the expectation that there will be no significant problems associated with skin sensitization.

Dexmethylphenidate, the chirally pure active isomer of methylphenidate, has been licensed as Focalin^R ^(Novartis) in the US in May 2005, also as an extended-release formulation [49,52]. As there is currently no evidence of superiority in terms of efficacy or tolerability [49,53], it has been assumed that Focalin^R ^will become available in Europe without a price premium over branded immediate-release (Ritalin^R^, also Novartis) or modified-release methylphenidate (Concerta^R^, Janssen-Cilag), respectively. Given its investment in Focalin^R^, it has been considered unlikely that Novartis would launch Ritalin^R ^LA (a long-acting preparation of methylphenidate available in the United States and some other markets) in England and Germany.

At the time of writing, few scientific data were in the public domain about lisdexamphetamine mesylate (NRP104), a new chemical entity that is an inactive prodrug of amphetamine believed to have a reduced potential for abuse and overdose compared to other ADHD drugs. The reason is that the amphetamine is conjugated to a specific amino acid and is activated only when metabolized in the gastrointestinal tract. Shire filed the product with the Food and Drug Administration (FDA) in December 2005 for the treatment of ADHD in children aged 6 to 12. Phase III study results presented at the Annual Meeting of the American Psychiatric Association in Toronto, May 2006, indicated similar efficacy and tolerability compared to mixed amphetamine salts (Adderall^R^) and duration of action of 12 hours [49]. A preliminary review of NRP104 by the U.S. Drug Enforcement Administration indicated that the compound would not be subject to controlled-substance scheduling [54], although this initial judgment has been revised since. The compound was launched by Shire in the United States end of July 2007 under the trade name "Vyvanse^R^". For modeling it was assumed that the product will be introduced to the English and German markets in 2008 and (except for the "Extremely Low Case" scenario) overtake modified-release methylphenidate products in terms of prescriptions by 2012. Obviously, this rests on the assumption that clinical advantages of NRP104 (regarding abuse potential, possibly tolerability profile, as well as label and summary of product characteristics) will be confirmed.

Modafinil has been licensed as "Provigil^R^" as a wake-promoting agent for patients with narcolepsy and shift work sleep disorder in the United States and England. Its manufacturer, Cephalon, has reformulated modafinil for children as once-daily 85 mg film-coated tablets, which it claims to be smaller and easier to swallow. Of note, this change of formulation should protect the new indication from generic competition [49]. While its mechanism of action is not fully understood, modafinil is classified as nonstimulant [55-57]. Similar to atomoxetine [58], a selective norepinephrine reuptake inhibitor, improvement of core symptoms had an effect size on core symptoms (school version of ADHD-RS) of 0.69 [59] to 0.76 [60]. This compares to effect sizes around 1.0 typically achieved with stimulants [58]. In August 2006, Cephalon announced discontinuation of modafinil development for ADHD due to safety concerns raised by the FDA, and the company intends to replace modafinil by its successor compound in development, armodafinil [61]. It has been assumed for modeling that armodafinil (like atomoxetine [62,63]) would become a second line treatment option after stimulants.

Finally, mixed amphetamine salts are marketed successfully in the U.S. (Adderall^R^, Adderall^R ^XR, by Shire) but have been licensed neither in England nor in Germany. It is believed that these products will not become available in Europe. Further compounds in phase II clinical development for ADHD include selective GABA-B receptor antagonists (SGS742, by Saegis, a privately held company with Novartis among its investors) and ampakine molecules (by Cortex). These projects have been excluded from the present study in light of their inherent uncertainty; statistically, attrition rates of compounds in clinical phase II are as high as 62% [64].

### 2. Projected budgetary impact from the perspectives of the German Statutory Health Insurance (SHI, GKV) and the National Health Service (NHS) in England

**Figure 3:**
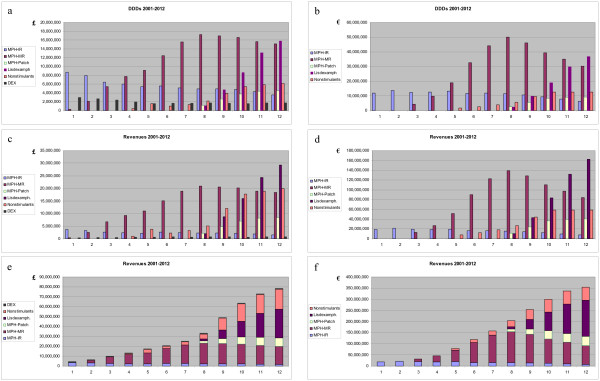
**Projected prescription drug expenditures for ADHD in children and adolescents, 2001 – 2012 (base case)**. a, b: Defined daily doses (DDDs) p.a.; c, d: expenditures by category p.a.; e, f: total (cumulated) expenditures p.a.; left: England; right: Germany. MPH: methylphenidate; IR: immediate-release formulations (Ritalin^R^, branded generics [Equasym^R^, Medikinet^R^], generics; Focalin^R^); MR: modified-release formulations (Concerta^R ^XL, Equasym^R ^XL, Medikinet^R ^retard, Focalin^R ^XR; MPH-Patch: transdermal system (Daytrana^R^); LisDEX: lisdexamphetamine (NRP104); Nonstimulants: atomoxetine (Strattera^R^), armodafinil (Nuvigil^R^); DEX: dexamphetamine (England only).

First, the model was calibrated using data on ADHD-related prescriptions and expenditures from 1998 to 2005 (cf. above). Besides a base case for projection through 2012 (Figure 3), four additional scenarios were defined to address the uncertainty surrounding critical assumptions (for details, see above and Table 4). Two scenarios (upper and lower bounds of base case) represented plausible variants, assuming different rates of treatment prevalence and intensity. Two further scenarios reflected extremes, the lower one assuming no price premiums for the new products and low treatment intensity, while the high extreme combined the effects of intense treatment with higher price premiums for new products.

**Table 4:** Key assumptions underlying scenarios (base case and extreme cases)

**Key assumptions^1^**	**Low Case (Extreme)**	**Base Case (Projection)**	**High Case (Extreme)**
Adjustments (all scenarios)	Germany: narcolepsy 2% of prescriptions in 2003; adult ADHD accounts for 10% of prescriptions in 2003 England: Provigil^R ^prescriptions cover narcolepsy exhaustively, no off-label use of modafinil for ADHD; modafinil for ADHD will be priced at the same level as Provigil^R^; adult ADHD accounts for 10% of prescriptions in 2003		
Peak diagnosis prevalence	England: 3.90% Germany: 4.20%	England: 3.90% Germany: 4.50%	England: 4.20% Germany: 4.80%
Peak treatment prevalence	England: 2.54% Germany: 3.15%	England: 2.54% Germany: 3.38%	England: 2.94% Germany: 3.84%
New product availability	dMPH (Focalin^R^) 2007; MPH-TDS 2008; LisDEX 2008; ARM/MOD ./.	dMPH (Focalin^R^) 2007; MPH-TDS 2008; LisDEX 2008; ARM/MOD 2009	
New products, specific notes	ARM/MOD *not *approved; LisDEX without clinical advantage over MPH-MR; dMPH (IR/MR) profile comparable to MPH-IR and MPH-MR08, respectively; MPH-TDS advantage limited to "convenience", no sensitization problems	ARM/MOD comparable to ATX; LisDEX: reduced abuse and diversion potential shown; dMPH (Focalin^R^) profile comparable to MPH-IR (dMPH-IR) and MPH-MR08 (dMPH-MR), respectively; MPH-TDS advantage limited to "convenience", no sensitization problems	ARM comparable to ATX; LisDEX: non-scheduled for reduced abuse potential; dMPH (Focalin^R^) profile comparable to MPH-IR and MPH-MR08, respectively; MPH-TDS advantage limited to "convenience", no sensitization problems
New product pricing	Focalin^R ^= branded MPH-IR; Focalin^R ^XR = MPH-MR12; LisDEX and MPH-TDS 50% premium over MPH-MR12; ARM/MOD n.a.	Price of dMPH-IR (Focalin^R^) = branded MPH-IR; dMPH-MR (Focalin^R ^XR) = MPH-MR12; LisDEX and MPH-TDS 50% price premium over MPH-MR12; ARM/MOD = ATX (20% b.i.d.) in Germany; ARM/MOD for ADHD in England priced like Provigil^R^.	Focalin^R ^= branded MPH-IR; Focalin^R ^XR = MPH-MR12; LisDEX and MPH-TDS 100% premium over MPH-MR12; ARM/MOD = LisDEX (D)
Established products	No price increases (except for DEX in England in "Extremely High Case" scenario). Generic MPH-IR market up to 90% (Germany) or 95% (England), respectively; Focalin^R ^up to 20% of MPH-IR market share. No generic substitution for ATX or MPH-MR market segments. ATX administered b.i.d. in 20% of patients.		
Treatment intensity	No change compared to 2005 (current trend ends 2006)	Continuation of current trend, phasing out by 2010	Continuation of current trend, phasing out by 2012

^1^Abbreviations: MPH: methylphenidate; IR: immediate-release formulations (Ritalin^R^, branded generics [Equasym^R^, Medikinet^R^], generics; Focalin^R^); MR: modified-release formulations (MPH-MR12: Concerta^R ^XL; MPH-MR08: Equasym^R ^XL, Medikinet^R ^retard, Focalin^R ^XR; MPH-TDS: transdermal system (patch, Daytrana^R^); LisDEX: lisdexamphetamine (NRP104); Nonstimulants: ATX, atomoxetine (Strattera^R^), ARM, armodafinil (Nuvigil^R^); DEX: dexamphetamine (England only)

While all scenarios will be described briefly below, Tables 4 and 5 have been limited to a more detailed account of the base case and the extreme scenarios. Interested readers may retrieve the full details from our Institute's website [97].

**Table 5:** Projected expenditures by scenario: ADHD pharmacotherapy for children and adolescents in England and Germany, 2012

**Key projections^1^**	**England**			**Germany**		
**Scenario**	**Low Extreme**	**Base Case**	**High Extreme**	**Low Extreme**	**Base Case**	**High Extreme**

**Expenditures 2012**	**49 m£**	**78 m£**	**117 m£**	**170 m€**	**311 m€**	**456 m€**
Increase 2012 over 2002	+602%	**+1,012%**	+1,561%	+617%	**+1,210%**	+1,825%
Total CNS^2 ^market (2012)	2,039 mŁ(1,463 mŁ)	**2,068 m£ (1,492 m£)**	2,107 mŁ(1,531 mŁ)	2,261 m€(1,585 m€)	**2,402 m€ **(1,726 m€)	2,547 m€(1,871 m€)
Share of total^2 ^market (2012)	2.4% (3.4%)	**3.8% (5.2%)**	5.5% (7.6%)	7.5% (10.7%)	**12.9% **(18.0%)	17.9% (24.4%)
**Projections by products**						
**Stimulants**	**40.7 m£**	**58.3 m£**	**91.3 m£**	**141 m€**	**259 m€**	**390 m€**
DEX	0.6 m£	**0.7 m£**	1.2 m£	n.a.	**n.a**.	n.a.
MPH-IR	1.2 m£	**1.5 m£**	1.9 m£	5 m€	**7 m€**	8 m€
MPH-MR	22.3 m£	**18.4 m£**	24.4 m£	90 m€	**74 m€**	92 m€
MPH-TDS	6.7 m£	**8.4 m£**	14.2 m£	24 m€	**36 m€**	58 m€
LisDEX	10 m£	**29.2 m£**	49.6 m£	22 m€	**143 m€**	232 m€
**Nonstimulants**	**8.6 m£**	**20.0 m£**	**25.5 m£**	**29 m€**	**51 m€**	**67 m€**
ATX	8.6 m£	**10.7 m£**	13.7 m£	29 m€	**39 m€**	47 m€
ARM	n.a.	**9.2 m£**	11.8 m£	n.a.	**13 m€**	20 m€

^1^For abbreviations, see legend to Table 4. ^2^(share of) market for psychotropics (D) or CNS drugs (UK), calculated assuming a growth rate of 5% p.a. (figures in brackets represent one of the sensitivity analyses, indicating market shares assuming no growth of non-ADHD market segment); for comparison: share of market segment in 2002 was 0.77% (NHS, England) and 1.8% (GKV, Germany), respectively.

The base case scenario, which is believed to reflect the most probable course of future events, implies an increase of drug spending for children and adolescents with ADHD for year 2012 by a factor of 12 (GKV in Germany) or 10 (NHS in England) over 2002. Assuming an annual growth rate of 5% for drug expenditures between 2005 and 2012 (except for ADHD in children and adolescents), then the projected ADHD treatment costs (from the NHS perspective, £78 million) would make up 3.8% of NHS spending for CNS drugs in 2012, compared to just 0.77% in 2002. For the German GKV (projected spending, €311 million), the corresponding figures would be 12.9% of total spending for psychotropic drugs in 2012, compared to 1.8% in 2002. This increase is driven by the multiplicative effects of increasing awareness and recognition of ADHD, growing rates of pharmacotherapy, somewhat increased intensity (dose and duration) of treatment, and the shift to novel, more expensive products (cf. Figure 3). The upper and lower bounds of the base case can be interpreted as sensitivity analyses with regard to treatment intensity; these indicate a plausible range of spending estimates from €259 to €380 million in Germany and from £63 to £101 million in England in 2012. The differences between both jurisdictions reflect the effects of differences in population size, unit costs, available products, and a lower baseline and less dynamic increase in England compared to Germany.

**Figure 4:**
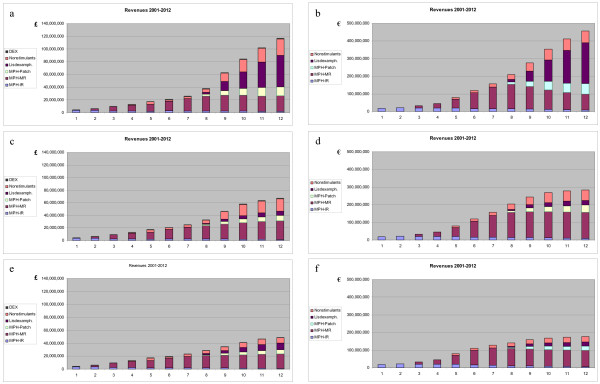
**Range of plausible projections: expenditures under extreme case scenarios, 2001–2012**. a, b: "High Extreme" Case; c, d: Base Case modified "LisDEX without clinical advantage over MPH-MR"; e, f: "Low Extreme" Case; left: England; right: Germany. MPH: methylphenidate; IR: immediate-release formulations (Ritalin^R^, branded generics [Equasym^R^, Medikinet^R^], generics; Focalin^R^); MR: modified-release formulations (Concerta^R ^XL, Equasym^R ^XL, Medikinet^R ^retard, Focalin^R ^XR; MPH-Patch: transdermal system (Daytrana^R^); LisDEX: lisdexamphetamine (NRP104); Nonstimulants: ATX, atomoxetine (Strattera^R^), ARM, armodafinil (Sparlon^R^); DEX: dexamphetamine (England only).

The extremely high scenario indicates the sensitivity of projections to higher prices of new products (cf. Tables 4 and 5). Additional scenarios were computed and fell within the range indicated. Of note, a scenario with much less successful lisdexamphetamine (assuming no advantage over modified-release methylphenidate) produced a spending projection of €249 million or £67 million in 2012, roughly corresponding to the lower bound of the base case (Figure 4). An extremely low case was calculated resting on very conservative assumptions (cf. Tables 4 and 5) regarding diagnosis and treatment prevalence rates, absence of any further increase in treatment intensity, a disappointing clinical profile and subsequent low adoption rate of lisdexamphetamine, and denial of market access for modafinil and armodafinil. This led to an estimated spending of €170 m or £49 million in 2012, still an increase over 2002 by a factor >6 for both Germany and England (Figure 4).

### 3. Impact on individual physicians

**Figure 5:**
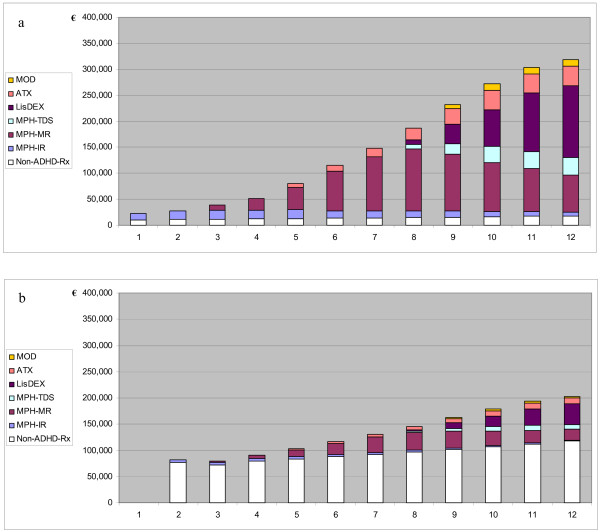
**Projected impact of ADHD treatment for children and adolescents on individual physicians' prescription drug expenditures, 2001–2012 (base case)**. a: Child and adolescent psychiatrists; b: pediatricians in private practice in Germany. Expenditures expressed as €/physician and year; perspective of statutory health insurance (i.e., excluding privately health insured patients). Data represent average values for one of the upper 50% of child and adolescent psychiatrists and one of the upper 20% of pediatricians in terms of relative involvement in care for ADHD patients, respectively. Concentration of care modeled according to Nordbaden data [74]. Abbreviation: Non-ADHD-Rx: expenditures for treatment of conditions other than ADHD.

These projections have been related to individual German physicians involved in care for children and adolescents with ADHD. Since ADHD care is a highly concentrated phenomenon (cf. Table 2), average spending (again, from the perspective of the GKV) for ADHD pharmacotherapy was calculated for the top-50% of child and adolescent psychiatrists, and the top-20% of pediatricians (see Figure 5).

Under base case assumptions, total annual drug spending of these child and adolescent psychiatrists would grow from an average total of less than €30,000 per physician in 2002 to €282,000 in 2012, of which €264,000 would be caused by prescriptions for ADHD treatment in children and adolescents (Figure 5). (Of course, as adult patients with ADHD have not been included in this analysis, the total budgetary impact of ADHD may be greater for those specialists who also treat adults, for example parents of patients.) This almost tenfold increase is the result of (a) the projected ADHD spending in combination with (b) the high concentration of patients among a small total number of specialists and (c) the very small number of prescriptions traditionally written by this specialist group. Even for the extremely low case looked at, this overall increase of drug spending by individual physicians would result in >5-fold drug spending caused by these specialists in 2012, compared to 2002. In this most conservative scenario, €144,500 or 89% of projected expenditures of €162,600 per physician in 2012 would still be accounted for by young ADHD patients.

Pediatricians as a group cause higher drug expenditures, and each of them treats a smaller number of ADHD patients compared to specialists, even those who are among the top-20% service providers for children with ADHD among their group. Hence, the incremental impact of ADHD-related spending is smaller compared to child and adolescent psychiatrists (Figure 4). Still, under base case conditions, ADHD-related pharmaceutical expenditures would account for 39% (or €75,000 out of €193,000 per physician in 2012, assuming a 5% growth rate of non-ADHD pharmaceutical spending) of total drug costs induced by this group. These figures compare with average drug costs of €83,000 per pediatrician in 2002. Thus, ADHD-related prescriptions alone would account for two thirds of the growth in drug expenditures caused by these physicians between 2002 and 2012.

## Discussion and limitations

Without exception, all plausible scenarios for England and Germany point to a further increase of expenditures for ADHD medication. The striking extent of the projected increase may appear counterintuitive, but it is easily explained by the simultaneous and multiplicative effects exerted by four variables, i.e., increases in diagnosis prevalence, treatment prevalence, treatment intensity, and the shift to more expensive new products. Over a wide range of assumptions, all projections concur indicating a strong impact of this increase on the individual drug budgets of physicians treating patients with ADHD.

Some important limitations of the present analysis warrant discussion. First, the present analysis does not purport to convey value judgments. It has little to nothing to say about the clinical appropriateness of the prescriptions analyzed; its mere focus is on their budgetary impact.

Second, there is substantial uncertainty around future events. Compounds in development may be discontinued, marketed drugs may be withdrawn because of serious adverse events, safety concerns may slow down diffusion of new products, and so on. The dynamic health policy environment is yet another factor. However, it would hardly be a prudent response to uncertainty to abandon planning and taking a proactive role in the pursuit of efficient service provision.

In strategic management, scenario analysis is a well-established tool to deal with environmental uncertainty [65]. The practice of scenario planning implicitly acknowledges that "best guesses" of future events may be wrong. Therefore, any scenario should not be confused with a forecast of the future. Multiple scenarios are pen-pictures of a range of plausible futures (Figures 3 and 4). Though each individual scenario has an infinitesimal probability of actual occurrence, combined a range of scenarios can shed light on possible future outcomes. This in turn should enable to plan for the range of futures that could, plausibly, unfold.

A third important limitation of this study concerns its restricted scope. Although pharmaceutical spending is likely to rise faster than other ADHD-related expenditures, it is certainly not the only cost component to be considered in a complete budgetary impact analysis [30,66]. Even more importantly, cost analyses illuminate just one half of the health economic equation; they do not provide information on "value for money", frequently analyzed in terms of cost-effectiveness [67]. Cost-effectiveness analyses of ADHD treatment strategies, however, have been rare [68], are only now beginning to appear [69-71], and have generally been limited to a one-year time horizon [72].

## Implications

In combination, the scenarios developed here strongly suggest that the trend of rising drug expenditures for ADHD may not abate in the near future (Figure 3).

They further indicate that physicians involved in providing care for children and adolescents with ADHD should anticipate escalating expenditures and challenging questions from payers (Figure 5). In particular for German child and adolescent psychiatrists, as well as for pediatricians in private practice, there will be a growing need to demonstrate appropriate prescribing practices and the achievement of clinical benefits commensurate with levels of spending.

Likewise, pharmaceutical manufacturers developing and selling products for the treatment of patients with ADHD will need to produce timely evidence supporting the economic value of their products.

Finally, it should be noted that the present study was limited to ADHD in children and adolescents age 6 to 18 years. There is reason to assume that adult ADHD may be associated with substantial spending dynamics, which has only just begun to show [73]. This fact, however, lends further support to the key conclusion of the present study, namely, that there will be a rapidly growing need for health economic evidence on the value of clinical interventions for ADHD.

## Abbreviations

ARM – armodafinil

ATX – atomoxetine

DEX – dexamphetamine

dMPH – dexmethylphenidate

FDA – Food and Drug Administration, the United States regulatory agency deciding on marketing authorizations for pharmaceutical products

GKV – Gesetzliche Krankenversicherung, statutory health insurance in Germany, covering approximately 90% of the population

IR – immediate release (formulation)

LisDEX – lisdexamphetamine, a prodrug of amphetamine in phase III clinical development

MAS – mixed amphetamine salts

MOD – modafinil

MPH – methylphenidate

MR – modified release (formulation)

MR08 – modified release formulation with duration of action of approximately 8 hours

MR12 – modified release formulation with duration of action of approximately 12 hours

NHS – National Health Service (England)

SHI – statutory health insurance in Germany (see GKV)

TDS – transdermal system
